# Metabolites Profiling of *Manilkara mabokeensis* Aubrév Bark and Investigation of Biological Activities

**DOI:** 10.1155/2022/4066783

**Published:** 2022-05-28

**Authors:** Xavier Worowounga, Rami Rahmani, Armel-Frederic Namkona, Sylvie Cazaux, Jean-Laurent Syssa-Magalé, Hubert Matondo, Jalloul Bouajila

**Affiliations:** ^1^Laboratoire de Génie Chimique, Université Paul Sabatier, CNRS, INPT, UPS, Toulouse, France; ^2^Laboratoire d'Analyse, d'Architecture et de Réactivité des Substances Naturelles (LAARSN), Faculté des Sciences, Université de Bangui, BP 908, Bangui, Central African Republic; ^3^Research Unit of Valorisation of Actives Biomolecules, Higher Institute of Applied Biology Medenine, University of Gabes, 4119, Medenine, Tunisia; ^4^Department of Life Sciences, Faculty of Sciences of Gabès, University of Gabès, Gabès, Tunisia

## Abstract

*Manilkara mabokeensis* Aubrév is a tree that belongs to the Sapotaceae family, native to the tropical forest in Latin America, Asia, Australia, and Africa. The bark of this species is used as traditional medicine to treat diseases. The present study is the first phytochemical investigation on *M. mabokeensis* Aubrév bark in terms of phytochemical composition and bioactivity. Among the different extracts, ethyl acetate (EtOAc) exhibited the highest values of total phenolic content (TPC), total flavonoid content (TFC), condensed tannin content (CTC), and reducing sugar content, as well as a high antioxidant activity. Interestingly, gas chromatography-flame ionization detector (GC-FID) and gas chromatography-mass spectrometry (GC-MS) analysis were enabled to identify 23 compounds in the essential oil of *M. mabokeensis* Aubrév bark, which have not been previously described in the literature. Phytol and 8,14-cedranoxide were the major identified compounds, with area percentages of 27.9 and 18.8%, respectively. For HPLC analysis, 3,4-dihydroxy-benzoic acid methyl ester showed the highest concentration with 61.8 mg/g of dry residue (dr) among other identified molecules. *Manilkara mabokeensis* Aubrév bark MeOH extract showed a good anti-15-lipoxygenase (anti-15-LOX) and anti-acetylcholinesterase (anti-AChE) activities of 65.8 and 71.0%, respectively, while it exhibited a moderate antixanthine oxidase (anti-XOD) activity (41.5%) at 50 *μ*g/mL. Furthermore, cyclohexane (CYHA) and ethyl acetate induced the highest cytotoxicity against the human ovarian cancer cell lines, OVCAR (49.5%) and IGROV (48.7%), respectively. Taken together, obtained results argue that *M. mabokeensis* Aubrév bark is an excellent source of natural compounds and justify its use in folk medicine.

## 1. Introduction

The Central African region is particularly highly endowed with diverse vegetation types. This region is the home of about 40.850 plant species, in which 6.000 are considered as endemic species, constituting tropical rainforests and coastal and alpine forests [1]. Nowadays, medicinal plants are attracting increasing attention. These plants have been known to have effective utilities and most of the rural population depends on them in primary healthcare [2]. Ethnomedicinal studies play a vital role to discover new drugs from indigenous medicinal plants. Sustainable pharmacy is getting more popular and vast opportunities for new drug discoveries are provided by the unmatched availability of chemical diversity and natural products, either as pure compounds or as homogenous plant extracts [3]. Therefore, plants have been used as source of remedies for the treatment of many diseases since ancient times and people of all continents, especially Africa, have this old tradition [4]. Medicinal plants are in great demand by the population thanks to their safety, effectiveness, lower costs, and biological activities.

The genus *Manilkar*a includes 135 plants that are distributed worldwide, of which about 20 can be found in tropical Africa [5]. These species can be the roots of different chemical compounds belonging to different classes, including phenolic acids, flavonoids, and terpenes [6]. This genus includes many species with economic and therapeutic importance. Bark is used to treat diarrhoea and dysentery, while the leaves are endowed by antimicrobial and antioxidant activities [7]. Moreover, the *Manilkar*a plant genus is recognized for its latex and chewing gum production [8]. *Manilkara mabokeensis* Aubrév, commonly known as Maboké sapodilla or Monghinza (Central African Republic), is a 25 m tall tree with a trunk diameter that can reach 100 cm [9]. This tree grows wild in the central African countries, especially Gabon and Central Africa [10]. In the past, *M. mabokeensis* Aubrév wood has been used in the construction of hunting elements (bow, crossbow, and trigger) [11]. To the best of our knowledge, there are no reports about the antioxidant and biological characterization of *M. mabokeensis* Aubrév bark, except our last work about the antimicrobial activity of *M. mabokeensis* Aubrév [12]. For that reason, this work was conducted to evaluate the chemical composition of organic extracts and essential oil, as well as the different biological activities (DPPH, 15-LOX, AChE, XOD, and cytotoxicity) of *M. mabokeensis* Aubrév bark.

## 2. Material and Method

### 2.1. Reagents

All chemicals used were of analytical reagent grade. All reagents were purchased from Sigma Aldrich (France): acetic acid, acetonitrile (ACN), cyclohexane (CYHA), dichloromethane (DCM), methanol (MeOH), Dulbecco's modified Eagle's medium (DMEM), dimethyl sulfoxide (DMSO), tamoxifen, Folin-Ciocalteu reagent (2 N), gallic acid, quercetin, catechin, HCl, KH_2_PO_4_, MTT, NaOH, sodium carbonate, 15-LOX, and AChE. Cell lines (OVCAR and IGROV) were purchased from American Type Culture Collection (USA).

### 2.2. Plant Collection

The bark of *M. mabokeensis* Aubrév (Sapotaceae) was collected from trees growing spontaneously in their natural habitats in October 2012. The trees originate from the region of Boukoko situated in the southwest of Central African Republic (CAR). The tree was identified using an authenticated herbarium and samples of its bark were dried in the laboratory at ambient temperature. A voucher specimen was deposited at the laboratory of Analysis, Architecture and Reactivity of Natural Substances (CAR), under the number of Mm012. The dried bark was ground using a mixer into fine powder.

### 2.3. Preparation of the Plant Extracts and Essential Oil

200 g of fine powder was successively extracted using 4 solvents of increasing polarity (CYHA, DCM, EtOAc, and then MeOH). A magnetic stirrer was used for extraction, which lasts 4 hours for each solvent. After filtration, each filtrate was evaporated using a rotary evaporator under vacuum at 35°C. The different residues obtained were evaluated for their chemical composition and their biological activity. For the essential oil, a hydrodistillation procedure was used. A mass of bark powder (50 g) was used for extraction by hydrodistillation using a Clevenger-type apparatus during 4 h in the Laboratory of Analysis, Architecture and Reactivity of Natural Substances (CAR).

### 2.4. Quantification of Total Phenolic Content (TPC)

The TPC of *M. mabokeensis* Aubrév bark extracts was determined using a Folin-Ciocalteu method, with minor modifications [13]. Briefly, the reaction mixture containing 20 *μ*L of diluted plant extract (0.5 mg/mL) and 100 *μ*L of Folin-Ciocalteu reagent (0.2 N) was left at room temperature for 5 min before adding 80 *μ*L of sodium carbonate (75 g/L in water). After 25 min of incubation at ambient temperature, the absorbance was measured at 765 nm, using a microplate reader (Multiskan Go, F1-01620, Finland). A standard calibration curve was obtained using gallic acid (0–115 *μ*g/mL). Results were expressed in milligram of gallic acid equivalents per g of dry residue (mg GAE/g dr).

### 2.5. Quantification of Total Flavonoids Content (TFC)

The TFC, in the various extracts, was estimated according to the Dowd method as described by Kohoude et al. [14]. A volume (100 *μ*L) of the diluted extract (0.5 mg/mL) was mixed with 100 *μ*L 2% solution of aluminum trichloride (AlCl_3_) in MeOH. After an incubation of 15 min, the absorbance was measured at 415 nm against blank sample (MeOH). Quercetin (2–10 mg/L) was used as reference compound to allow drawing the standard curve. The results were expressed in milligram of quercetin equivalents per g of dry residue (mg QE/g dr).

### 2.6. Quantification of Total Condensed Tannin Content (CTC)

The determination of the CTC in the different extracts was done using the same procedure described by Kohoude et al. [14]. The diluted solution of each extract (50 *μ*L) was mixed with vanillin (1% in 7 M H_2_SO_4_, 100 *μ*L) in an ice bath. After that, the mixture was shaken and incubated at ambient temperature for 15 min. The absorbance of the samples was measured at 500 nm. Catechin (3–16 mg/L) was used as reference to make the calibration curve. The results were expressed in milligram of catechin equivalents per g of dry residue (mg CE/g dr).

### 2.7. Quantification of Total Anthocyanins Content (TAC)

The TAC contained in the various extracts of *M. mabokeensis* Aubrév bark was determined using the pH differential absorbance method as described by Kohoude et al. [14]. Two buffer solutions were prepared: The first solution consisted of hydrochloric acid (pH = 1.0) and potassium chloride (0.2 M). The second buffer solution was a mixture of acetic acid (pH 4.5) and sodium acetate (1 M). Briefly, 180 *μ*L of the buffer solution was added to 20 *μ*L of extract. The reading was made on two wavelengths at 510 and 700 nm after 15 min of incubation. The following equation was applied for the calculation: A = [(A_510_-A_700_)_pH 1.0_-(A_510_-A_700_)_pH 4.5_]. The results were expressed in milligram of cyanidin-3-glucoside equivalents per g of dry residue (mg C3GE/g dr).

### 2.8. Quantification of Reducing Sugar Content

The sugar content quantification of *M. mabokeensis* Aubrév bark extract was done according to the procedure used by Kouhoude et al. [14], with minor modifications. Briefly, 100 *μ*L of each extract at 5 mg/mL was mixed with 150 *μ*L of DNS solution (0.05 M). Then, 750 *μ*L of deionised water was added after stirring and incubation for 5 min in a bath-husband at 100°C. After a second stirring, the absorbance of the mixture was measured at 530 nm against a blank consisting of the same sample, in which the DNS was replaced by 5% DMSO, and against a negative control wherein the extract was replaced by 5% DMSO. The amount of sugar was determined in mg of glucose equivalent per gram of dry residue (mg GAE/g dr).

### 2.9. Chromatographic Analysis

#### 2.9.1. Essential Oil Analysis

The chemical identification and quantification of *M. mabokeensis* Aubrév bark essential oil were done following the procedures of Kohoude et al. [14]. Gas chromatography-flame ionization detection (GC-FID) analyses were carried out on a Varian Star 3400-x chromatograph (France) fitted with a fused silica capillary DB-5MS column (5% phenylmethylpolysiloxane, 30 × 0.25 mm, film thickness 0.25 *μ*m). Chromatographic conditions were, firstly, from 60 to 260°C, and then the temperature rose with a gradient of 5°C/min and 15 min isotherm at 260°C. After that, a second gradient was applied to 340°C at 40°C/min. For analysis reasons, petroleum ether was used to dissolve the essential oil. One microliter was injected in the split mode ratio of 1 : 10 and the helium was used as carrier gas at 1 mL/min. The injector was operated at 200°C. For the gas chromatography-mass spectrometry (GC-MS) system (Varian Saturn 2000 ion trap GC/MS with CP-3800 GC), it was used with the same chromatographic conditions as GC-FID. The MS system was adjusted for an emission current of 10 *μ*A and electron multiplier voltage between 1400 and 1500 V. The trap temperature was 250°C and that of the transfer line was 270°C and the mass scanning was from 40 to 650 amu. The identification of the compounds was done by (i) comparison of their retention index (RI) relative to C5–C24 n-alkanes obtained on a nonpolar DB-5MS column, with those provided in the literature, and (ii) comparison of their mass spectra with those recorded in NIST 08, reported in published articles or by coinjection of available reference compounds. The percentage composition of the essential oil was measured by the normalization method from the GC peak areas, assuming identical mass response factor for all compounds.

#### 2.9.2. Organic Extracts Analysis

The volatile compounds identification from the different organic extracts, before or after derivatization, was carried out with the same equipment GC-MS. The analysis was done following this gradient: 5 min at 60°C, then 60–270°C at 15°C/min, 6 min at 270°C, 270–300°C at 50°C/min, and finally 300°C at 4.5 min. The entire chromatographic program lasted 30 min. The derivatization method was that described by Kohoude et al. [14], with minor modifications.

#### 2.9.3. Phenolic Compounds Analysis by HPLC-DAD

The HPLC analysis was performed in an UltiMate 3000 Pump-Dionex and Thermo Separation Products detectors UV-150 model (Thermo Fisher Scientific, USA) as mentioned by Rahmani et al. [15]. The separation was done on an RP-C18 column (25 cm × 4.6 mm, 5 *μ*m) at ambient temperature (20–25°C). Elution was performed at a flow rate of 1.2 mL/min, using a mobile phase consisting of acidified water (pH = 2.65) (solvent A) and acidified water/ACN (20 : 80 v/v) (solvent B). The samples were eluted by the following linear gradient: from 0.1 B to 30% B for 35 min, from 30 B to 50% B for 5 min, from 50 B to 99.9% B for 5 min, and finally from 99.9 B to 0.1% B for 15 min. The extracts were prepared at the concentration of 20 mg/mL using the mixture acidified water/ACN (80 : 20 v/v) and then filtered by a filter (Sigma-Aldrich, Millex-HA filter 0.45 *μ*m, France). Then, 20 *μ*L of each sample was injected and the detection was done at 280 nm. The phenolic compounds were identified by comparison of the retention time of some known standards and then quantified using their calibration curves (3-amino-4-hydroxybenzoic acid; gallic acid; 9-chloro-10-hydroxy-2,3-dimethyl-anthracene-1,4-dione; brilliant yellow; hamamelitannin; 3,4-dihydroxy-5-methoxybenzoic acid; chlorogenic acid; (−)-epicatechin; 3,4-dihydroxycinnamic acid; 3,4-dihydroxy-5-methoxycinnamic acid; dihydromyricetin; 2,4-dihydroxycinnamic acid; methyl 3,5-dihydroxybenzoate; 6-hydroxycoumarin; 4,7-dihydroxycoumarin; 3,4-dihydroxy-benzoic acid methyl ester; 7-hydroxycoumarin; 7-hydroxycoumarin-3-carboxylic acid; 7-hydroxycoumarin-3-carboxylic acid; N-succinimidyl ester; 4-hydroxy-3-methoxycinnamic acid; gallocyanine; rutin hydrate; 7-hydroxy-6-methoxycoumarin; polydatin; sinapic acid; chicoric acid; taxifolin hydrate; myricitrin dehydrate; taxifolin; quercetin 3-*β*-D-glucoside; *α*-cyano-4-hydroxycinnamic acid; trihydroxyethylrutin; 5,3′-dihydroxyflavone; salvianolic acid B; 3′-hydroxy-a-naphthoflavone; 9-chloro-10-hydroxy-anthracene-1,4-dione; coumarin; ethyl 3,5-dihydroxybenzoate; rhapontin; isopropyl 3,4,5-trihydroxybenzoate; 2-hydroxycinnamic acid; 3-cyano-7-hydroxycoumarin; diosmin; rosmarinic acid; myricetin; 2,4-dihydroxy-3,6-dimethylbenzoic acid; 3-cyano-7-hydroxy-4-methylcoumarin; methyl 4-hydroxybenzoate; *trans*-cinammic acid; 1,3-dihydro-5,6-dimethoxy-3-[(4-hydroxyphenyl)methylene]-H-indol-2-one; 7,3′-dihydroxyflavone; 5,8-dihydroxy-1,4-naphthoquinone; icariin; ethyl 3,4-dihydroxycinnamate; chrysin; 3',5′-dihydroxyflavone; 7,8-dihydroxy-2,2-dimethylchromane-6-carboxylic acid; wedelolactone; 5,7-dihydroxy-4-propylcoumarin; (E/Z)-endoxifen hydrochloride hydrate; butyl gallate.; 5-hydroxy-7-((3-methylbenzyl)oxy)-2-phenyl-4h-chromen-4-one; 4-hydroxytamoxifen; silibinin; 3-chloro-7-hydroxy-4-methylcoumarin; 4-ethyl-7-hydroxy-8-methyl-2H-chromen-2-one; 5,7-dihydroxy-4-phenylcoumarin; (z)-3-(3-ethoxy-4-hydroxy-phenyl)-2-phenyl-acrylic acid; cardamonin; 7-hydroxyflavone; phenoxodiol; baicalein; 3-tert-butyl-4-hydroxybenzoic acid; pinostilbene hydrate; 3-benzyloxy-4,5-dihydroxy-benzoic acid methyl ester; 6-hydroxyflavone; 7-hydroxy-4-(trifluoromethyl)coumarin; 6-hydroxy-4′-methylflavone; pinosylvin; 2-chloro-3-(4-hydroxy-phenylamino)-(1,4)naphthoquinone; ethyl trans-2-hydroxycinnamate; 7-hydroxy-4-methyl-3-coumarinylacetic acid; 7-hydroxy-4-phenylcoumarin; CU-CPT22; 3′-hydroxy-6-methylflavone; caffeic acid 1,1-dimethylallyl ester; 2-chloro-3-(2-hydroxy-5-methylanilino)-1,4-naphthoquinone; 4-hydroxy-3-propylbenzoic acid methyl ester; 7-hydroxy-3′,4',5′-trimethoxy-alpha-naphthoflavone; plumbagin; 4′,5-dihydroxy-7-methoxyflavone; butyl 4-hydroxybenzoate; benzyl 4-hydroxybenzoate; 3,3',4'-trimethoxyflavone; isobutyl 4-hydroxybenzoate; diethylstilbestrol; combretastatin A4; 4-hydroxy-3-(3-oxo-1-phenylbutyl)coumarins; cinnamyl-3,4-dihydroxy-*α*-cyanocinnamate; pinosylvin monomethyl ether; 3,7-dimethoxyflavone; 3,3′-dimethoxyflavone; 2,3-dichloro-5,8-dihydroxy-1,4-naphthoquinone; 3,6,3′-trimethoxyflavone; shikonin; 10-[(3-hydroxy-4-methoxybenzylidene)]-9(10H)-HMBA; 5-hydroxyflavone; 5-hydroxy-3′-methoxyflavone; and 3′-hydroxy-b-naphthoflavone).

### 2.10. Biological Activities

#### 2.10.1. Determination of DPPH Radical Scavenging Activity

The free radical 2,2-diphenyl-1-picrylhydrazyl (DPPH) was used for antioxidant activity by applying the method used by Rahmani et al. [13], with some modifications. 20 *μ*L of various dilutions of the plant extract was mixed with 180 *μ*L of a 0.2 mM DPPH solution (dissolved in MeOH). The incubation lasted 30 min at 25°C, and the absorbance was measured at 520 nm. A_blank_ was measured without extract. DPPH inhibition was calculated as % inhibition = 100 x (A_blank_ - A_sample_)/A_blank_.

#### 2.10.2. Anti-15-Lipoxygenase Activity (15-LOX)

Human 15-LOX (from soybean) is the crucial enzyme that catalyzes the formation of bioactive leukotrienes (LT4A) from arachidonic acid (biological substrate) [15]. In this experiment (*in vitro*), linoleic acid (substrate) was oxidized to conjugate diene by 15-LOX. Briefly, 20 *μ*L of diluted extract (0.5 mg/mL) was mixed with 170 *μ*L of Na_3_PO_4_ buffer (pH = 7.4), 60 *μ*L of linoleic acid, and 20 *μ*L of enzyme solution (15-LOX). The absorbance was measured at 234 nm. A_blank_ was measured without extract. Nordihydroguaiaretic acid (NDGA) was used as positive control. The enzyme activity inhibition was calculated as follows: % inhibition = 100 x (A_blank_ – A_sample_)/A_blank_.

#### 2.10.3. Antiacetylcholinesterase Activity (AChE)

The AChE activity was determined using the Ellman colorimetric method as previously described by Kohoude et al. [14]. Quickly, 50 *μ*L of 0.1 mM sodium phosphate buffer (pH = 7.5), 125 *μ*L of DTNB, 25 *μ*L of diluted plant extract (0.5 mg/mL), and 25 *μ*L of enzyme solution were mixed and incubated for 15 min at 25°C. Subsequently, 25 *μ*L of ACTHi was added, and then the final mixture was incubated for 25 min at 25°C and the absorbance was measured at 421 nm. A_blank_ was measured without extract. The reference used was galanthamine. The enzyme activity inhibition percentage was calculated as follows:

(1) $$\begin{array}{c} \%\text{ inhibition}=100\times \frac{\left(A_{\text{blank}}\;-\;A_{\text{sample}}\right)}{A_{\text{blank}}}. \end{array}$$

#### 2.10.4. Xanthine Oxidase Inhibition Assay (XOD)

The XOD activity using xanthine as the substrate was evaluated using the procedure of Kohoude et al. [14]. The substrate solution (1 mM) was prepared by dissolving xanthine in 25 mL of 0.1 mM sodium phosphate buffer (pH = 7.5). The xanthine oxidase enzymatic solution was prepared by diluting xanthine oxidase enzyme from cow's milk (1 U) to a final concentration of 0.1 U/mL. The assay mixture consisted of 50 *μ*L of diluted plant extract (0.2 mg/mL), 60 *μ*L of 70 mM sodium phosphate buffer (pH = 7.5), and 30 *μ*L of the enzymatic solution, giving a final extract concentration of 50 *μ*g/mL in each well of a 96-well microplate. After an incubation of 25 min, 60 *μ*L of substrate solution was added and the absorbance was measured at 295 nm after 5 min. A_blank_ was measured without extract. Allopurinol was used as a positive control. The XOD activity was expressed as percent inhibition of XOD enzyme, calculated as follows: % inhibition = 100 x (A_blank_ - A_sample_)/A_blank_.

#### 2.10.5. Cytotoxicity Evaluation

The cytotoxicity of the different *M. mabokeensis* Aubrév bark extracts was estimated on IGROV and OVCAR cells lines (American Type Culture Collection) as described by Kohoude et al. [14], with minor modifications. Cells were distributed in 96-well plates at 3 × 10^4^ cells/well in 100 *μ*L. After that, 100 *μ*L of the corresponding culture medium (DMEM) containing sample at various concentrations was added. Cell growth was estimated by the MTT assay. MTT is a water-soluble tetrazolium salt with a yellow coloration. Metabolically active cells are able to convert the dye to water-insoluble dark blue formazan by reductive cleavage of the tetrazolium ring. The extracts were resolubilized in the DMSO followed by dilution in the buffer, whereby the DMSO does not exceed 1%. Doxorubicin was used as a positive control. The cells activity inhibition percentage was calculated as follows:

(2) $$\begin{array}{c} \%\text{ inhibition}=100\times \frac{\left(A_{\text{blank}}\;-\;A_{\text{sample}}\right)}{A_{\text{blank}}}. \end{array}$$

### 2.11. Statistical Analysis

All measurements were carried out in quadruplicate. One-way analysis of variance (ANOVA) was used for the significance calculation using the Statistical Package for the Social Sciences (SPSS) 20.1 (IBM, Version. 20.0.2004). Statistical differences between the solvents used in the study were estimated using Tukey's test. The determination of the relationship between TPC, TFT, TAC, CTC, and biological activities was assessed by the linear coefficient of determination (*r*; Pearson correlation coefficients). Principal component analysis (PCA) was performed using XLSTAT (version 2014.5.03) for visualization of discrimination between different parameters.

## 3. Results and Discussion

### 3.1. Extraction Yields and Chemical Composition of Extracts

The extraction yield, the sugar content, the total phenolic content (TPC), total flavonoid content (TFC), condensed tannin content (CTC), and total anthocyanin content (TAC) in the *M. mabokeensis* Aubrév bark extracts are shown in Table 1. According to the literature, no studies have been reported previously on the effect of solvents on extraction yield of *M. mabokeensis*. In this study, four different solvents, namely, CYHA, DCM, EtOAc, and MeOH, were used for the extraction. The yields of the extracts obtained varied from 0.8 to 25.0%, according to the solvent used. For all the tested extracts, MeOH extract gave the highest yield (25%), followed by CYHA extract (1.7%), then EtOAc (1.2%) extract, and finally DCM extract (0.8%) (Table 1). In general, the yield of the polar extract (MeOH) was twenty times higher than the yield obtained with DCM and was about fifteenfold greater than those of CYHA and EtOAc solvents. This difference of yields between the extracts could be due to the difference in chemical composition of extract, which allows us to suggest that the various extracts may contain more polar compounds. The present results were within the range of those found by Monisha et al. [16], when working on the bark extracts of *M. hexandra*. They confirm that the highest yield was found in the polar solvents (water and MeOH). The contents of reducing sugars (mg/g dr) in various extracts of *M. mabokeensis* varied according to the polarity of solvents used. While the CYHA and DCM extracts showed no reducing sugar content, EtOAc and MeOH ones revealed contents of 78.7 and 101.1 mg GAE/g dr, respectively (Table 1).

According to the literature, the mentioned amounts of chemical family of *M. mabokeensis* Aubrév bark are evaluated for the first time. Statistically, bark extracts of *M. mabokeensis* showed no significant difference between CYHA and DCM solvents compared to the EtOAc and MeOH ones in terms of their TPC. The EtOAc extract had the TPC in the samples (354.2 mg GAE/g dr), whereas CYHA and DCM extracts contained a small amount of TPC, with 0.9 and 10.2 mg GAE/g dr, respectively. The present results were in accordance with those found in the different extracts of *M. rufula* stem bark. Similarly, the EtOAc extract had the highest TPC compared to the other extracts with different polarities [17]. The current results showed a high TPC compared to *M. zapota* ethanolic extract as found by Hilma et al. [18]. They found a TPC that did not exceed 15 mg GAE/g dr.

While two extracts (EtOAc and MeOH) were found to exhibit flavonoids content, three extracts (DCM, EtOAc, and MeOH) highlighted tannin content. Statistically, there was a significant difference between the different extracts in terms of both TFC and CTC (Table 1). The condensed tannin content ranged between 2.0 and 9.9 mg CE/g dr, while the TFC did not exceed the 8.0 mg CE/g dr. In addition, the results showed that *M. mabokeensis* bark contained traces of anthocyanins. Among all tested extracts, only CYHA extract exhibited a low TAC of 0.03 C3GE/g dr (Table 1).

### 3.2. Chromatographic Analysis

#### 3.2.1. Identification of Volatile Compounds of the Essential Oil

In Table 2, the percentages of the compounds identified in the essential oil from the bark of *M. mabokeensis* are listed in sequence of their retention indices (RI). In total, 23 compounds were detected and identified, representing 99.92% of the total oil with 2.55% monoterpene hydrocarbons, 2.03% oxygenated monoterpenes, 21.27% sesquiterpene hydrocarbons, 42.94% oxygenated sesquiterpenes, and 31.13% others. This is the first report to investigate the chemical composition of the *M. mabokeensis* Aubrév bark essential oil and all the identified molecules were detected for the first time in *Manilkara* genus. The main constituents were found to be phytol and 8,14-cedranoxide, which were the major compounds, with area percentages of 27.19 and 18.88%, respectively. Other main components included two sesquiterpene oxygenated (caryophylleneoxide and 10-epi-*γ*-eudesmol) and three sesquiterpene hydrocarbon (*α*-gurjunene, cyclosativene, and *cis*-thujopsene) with area percentages of 5.86, 5.84, 7.39, 6.54, and 4.79%, respectively (Table 2). The number of monoterpenes was minor, compared to the sesquiterpene, with only six identified compounds. Eucalyptol and *α*-fenchene exhibited the highest area percentages of 1.56 and 0.81%, respectively.

#### 3.2.2. Identification of Volatile Compounds of the Different Extracts by GC-MS

The volatile compounds of *M. mabokeensis* Aubrév bark extracts were identified by GC-MS. No volatile compounds were detected in the EtOAc and MeOH extracts without derivation. However, four volatile compounds were detected in the CYHA and DCM ones. These compounds were distributed as follows: *trans*-sesquisabinene hydrate and cedrene in the CYHA extract and 8-cedren-13-ol detected in the DCM one. Meanwhile, *α*-copaene compound was detected in both extracts (Table 3). Trying to identify more volatile compounds in the different extracts, a derivatization reaction was used. Overall, 10 volatile compounds were identified in the different organic extracts (Table 3). Only one derivatized compound (phenol,2-(1,1-dimethylethyl)-6-methyl-) was observed in the DCM extract. On the other hand, this step led to the identification of 3 volatile compounds in both CYHA and EtOAc extracts and 5 compounds in the MeOH one. All the compounds were detected only in one extract. However, catechin was detected in both EtOAc and MeOH. According to the literature, the GC-MS analysis before and after derivatization is the first such study of the extracts of a plant of the *Manilkara* genus and none of the identified molecules have been previously mentioned in the literature for this plant.

#### 3.2.3. Identification and Quantification of the Phenolic Compounds by HPLC-DAD

**Figure 1:**
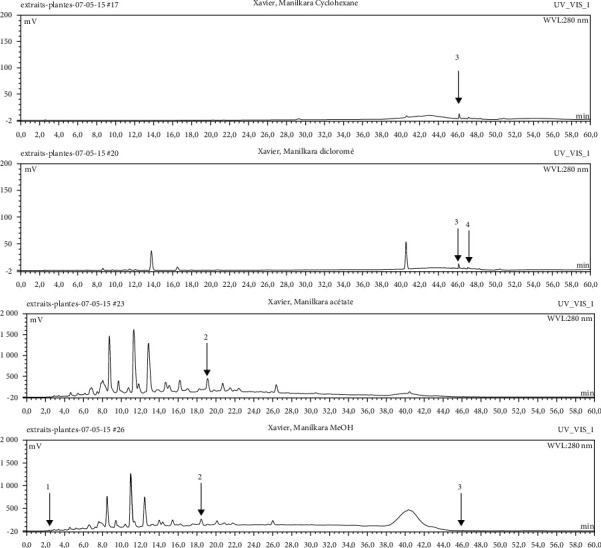
HPLC chromatograms of *M. mabokeensis* Aubrév bark extracts. CYHA: cyclohexane; DCM: dichloromethane; EtOAc: ethyl acetate; MeOH: methanol. Peaks: (1) 3-amino-4-hydroxybenzoic acid; (2) L-tyrosine 7-amido-4-methylcoumarin; (3) 4-hydroxy-3-propylbenzoic acid methyl ester; (4) 3,6,3′-trimethoxyflavoneIcariin.

HPLC-DAD analysis was done for the identification of phenolic compounds in bark extracts of *M. mabokeensis* Aubrév. The results of analysis led to the identification of only 4 phenolic compounds in all extracts by comparison of their relative retention time with those of standards with known retention time (Table 4; Figure 1). The concentrations of the identified compounds ranged from 0.06 to 61.76 mg/g dr. The compound 3,4-dihydroxy-benzoic acid methyl ester [20] was found in EtOAc and MeOH extracts with concentrations of 61.8 and 1.9 mg/g dr, respectively. While 4-hydroxy-3-propylbenzoic acid methyl ester [21] compound was detected in three different extracts (CYHA, DCM, and MeOH) with the same concentration (0.3 mg/g dr), the other two compounds (3-amino-4-hydroxybenzoic acid [19] and 3,6,3′-trimethoxyflavone [22]) were found only in one extract (Table 4). According to the literature, none of the identified compounds were found previously in *Manilkara* species.

### 3.3. Biological Activities

This is the first study to investigate the antioxidant, anti-15-LOX, anti-AChE, anti-XOD, and cytotoxic activities of *M. mabokeensis* extracts.

#### 3.3.1. Antioxidant Activity

**Figure 2:**
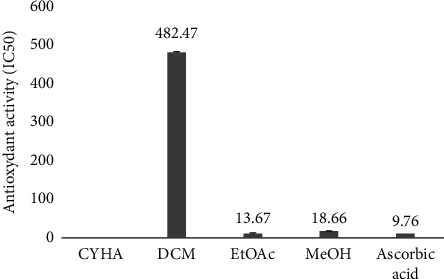
Antioxidant activity (IC50 mg/L) of *M. mabokeensis* Aubrév bark extracts by DPPH assay. CYHA: cyclohexane; DCM: dichloromethane; EtOAc: ethyl acetate; MeOH: methanol.

The antioxidant activity of *M. mabokeensis* Aubrév bark extracts was evaluated by the anti-free radical test (DPPH). Statistically, there was a significant difference (*p* ≤ 0.05) between the different extracts. EtOAc and MeOH extracts showed IC_50_ values of 13.7 and 18.7 *μ*g/mL, respectively (Figure 2). However, the DCM extract showed poor activity with IC_50_ = 482.5 *μ*g/mL and the CYHA extract was not active. Pearson's correlation coefficient, presented in Table 5, was determined by correlating the antioxidant activity inhibition and total amount of secondary metabolite classes. There was a low negative correlation between TPC-anti-DPPH, TFC-anti-DPPH, CTC-anti-DPPH, and TAC-anti-DPPH, with *r*-values of −0.51, −0.55, −0.38, and −0.36, respectively (Table 5). These findings indicate, probably, that the antioxidant effect of the different extracts could be due to other compounds besides the phenolic one. The present results were better than those found by Dutta and Ray [23]. In this study, the MeOH extract of *M. hexandra* bark showed an IC_50_ of 88.7 *μ*g/mL. Furthermore, a recent study, conducted by Chunhakant and Chaicharoenpong [24] showed that the IC_50_ of the MeOH and the aqueous bark extract of *M. zapota* were 66.4 and 78.0 *μ*g/mL, respectively. Therefore, its antioxidant potency was less important than that of the bark MeOH extract of *M. mabokeensis* Aubrév (IC_50_ : 18.7 *μ*g/mL). Moreover, a previous study showed that some compounds, such as taraxerol and taraxerone extracted from *M. zapota* bark, have a high antioxidant activity and an IC_50_ close to that in the current study [25].

#### 3.3.2. Anti-15-LOX Activity

*Manilkara mabokeensis* Aubrév bark extracts showed an anti-15-LOX inhibition effect ranked from 0 to 65.8% for the bark. Statistically, there was a significant difference (*p* ≤ 0.05) between the different extracts, regardless of the used solvent, in terms of 15-LOX inhibition. The polar extract (MeOH) showed the highest inhibition percentage of 65.8%, while the nonpolar (DCM and EtOAc) ones showed a moderate inhibition that did not exceed 25.7% (Table 6). No previous studies have investigated the anti-inflammatory activity of the *Manilkara* species extracts and the inhibition of the 15-LOX. In addition, Pearson's correlation coefficient presented in Table 5 showed a positive correlation between the group of phenolic compounds (TPC, CTC, and TFC) and the 15-LOX inhibition with *r*-values of 0.59, 0.95, and 0.74, respectively (Table 5). It is possible that the observed correlation was due to the considerably high amounts of phenolic compounds in polar extracts. Furthermore, this suggests that the phenolic metabolites are probably responsible for the bioactivity of extracts [26].

#### 3.3.3. Anti-AChE Activity

The AChE enzyme is involved in the hydrolysis of the neurotransmitter acetylcholine, which contributes to the pathogenesis of Alzheimer's disease [27]. All the extracts, except the CYHA one, showed an anti-AChE activity. Statistically, there was no significant difference (*p* > 0.05) between DCM and EtOAc extracts compared to the MeOH one, in terms of anti-AChE inhibition. The MeOH extract showed the potent activity against AChE enzyme with an inhibition of 71.0%. The other extracts (DCM and EtOAc) showed moderate inhibition activities of 43.5 and 46.5%, respectively (Table 6). Previous studies suggested that there is a correlation between TPC and AChE inhibition activity [28], which is the case in this study. According to the correlation matrix, a good correlation was found between TPC-anti-AChE, TFC-anti-AChE, and CTC-anti-AChE with *r*-values of 0.61, 0.69, and 0.72 (Table 5).

#### 3.3.4. Anti-XOD Activity

The xanthine oxidase enzyme is a flavoprotein that catalyzes the oxidation of hypoxanthine to uric acid. Therefore, anti-XOD activity was evaluated as the formation of uric acid from xanthine. Among all tested extracts, EtOAc and MeOH extracts showed an anti-XOD activity, with inhibition percentages of 11.1 and 41.5, respectively (Table 5). The other extracts showed no anti-XOD activity (Table 6). Statistically, as shown in the correlation matrix, there was a significant modest correlation between TPC and anti-XOD activity (*r* = 0.56) and between CTC and anti-XOD activity (*r* = 0.59), while this correlation was high between TFC and anti-XOD activity (*r* = 0.71) (Table 5). These results indicate that the flavonoids were the most potent compounds, which induce an anti-XOD activity [29].

#### 3.3.5. Cytotoxic Activity

The cytotoxic activity of *M. mabokeensis* extracts was assessed against two cell lines (OVCAR and IGROV) at 50 *μ*g/mL. While the EtOAc extract showed the most potent cytotoxic activity against IGROV with an inhibition percentage of 48.7%, the CYHA extract was more active against OVCAR cell line (49.5%) (Table 7). Statistically, the cytotoxicity of the different extracts depends on the solvent polarity. Pearson's correlation coefficient was determined by correlating the cytotoxicity against cancer cell lines and total amount of secondary metabolite classes. Thus, there was a high positive correlation between the TPC, TFC, CTC, and cytotoxic activity for IGROV cell line with *r*-values of 0.94, 0.91, and 0.86, respectively. However, only TAC was found to have a very high correlation with the cell line OVCAR (*r* = 0.99) (Table 5). These findings made it possible to suggest the involvement of a set of phenolic compounds in the inhibition of the IGROV cells growth. Nevertheless, anthocyanins were the most responsible compounds in the inhibition of OVCAR cell line [30].

### 3.4. Principal Component Analysis

**Figure 3:**
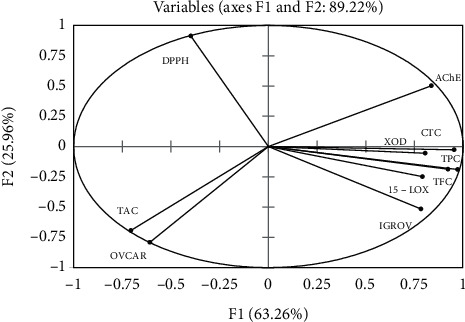
Principal component analysis “loading plot” of antioxidant properties (TPC: total phenolic content; TFC: total flavonoids content; TAC: total anthocyanins content; CTC: total condensed tannin content; DPPH: antioxidant activity) and biological activities (AChE: antiacetylcholinesterase activity; 15-LOX: anti-15-lipoxygenase activity; IGROV and OVCAR, and cytotoxic activity) of *M. mabokeensis* Aubrév bark extracts.

**Figure 4:**
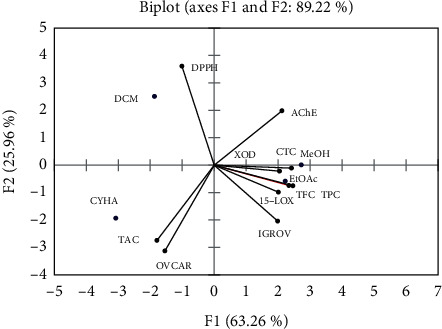
Principal component analysis “biplot” of biological activities *M. mabokeensis* Aubrév bark extracts.

Principal component analysis (PCA) was performed to understand how the TPC, TFC, TAC, and the CTC contribute to the different biological activities (anti-DPPH, anti-XOD, anti15-LOX, and cytotoxicity) of the plant extracts. Principal components (PC) 1 and 2 have eigenvalues of 6.33 and 2.60, respectively. PC1 and PC2 showed 63.26 and 25.96% of the total data variance, respectively, and both contribute 89.22% to the total variation (Figure 3). While PC2 showed, only, a strong positive correlation with the antioxidant activity (anti-DPPH) with a factor loading of 0.91, PC1 showed a strong positive correlation with the level of phenolic profile (TPC, TFC, and CTC) with a loading of 0.92, 0.97, and 0.95, respectively, with this being less pronounced for the variables, anti-AChE (*r* = 0.84), anti-XOD (*r* = 0.81), anti-15-LOX (*r* = 0.79), and cytotoxic activity (IGROV) (*r* = 0.78) (Table 8). OVCAR and TAC showed a negative correlation with both PC1 and PC2, which means that these two variables were correlated, probably, with the third axis (PC3). Overall, there was a strong positive correlation between OVCAR and TAC. TPC, TFC, and CTC positively contributed to an increase of potential inhibition against IGROV with Pearson correlation coefficients (*r*) equal to 0.94, 0.91, and 0.86, respectively. Finally, there was also a good correlation between XOD and 15-LOX having an *r*-value (Pearson correlation coefficients) of 0.98 (Table 5). The biplot figure (Figure 4) showed, firstly, that extracts were located, relative to TPC, TFC, TAC, and the different biological activities, on the basis of their chemical composition. Secondly, while EtOAc and MeOH were placed close to each other, CYHA and DCM were located separately (Figure 4). Overall, EtOAc and MeOH extracts were located close to the majority of variables (TPC, TFC, CTC, 15-LOX, XOD, IGROV, and AChE). This indicates that the polyphenol compounds contribute to the inhibition of these mentioned activities. However, CYHA extract, which contained the highest content of TAC, was responsible for the cytotoxic activity against OVCAR cell line.

## 4. Conclusion

In this study, evaluation on the chemical composition and biological activity of the plant *M. mabokeensis* was carried out as well as a series of phenolic compounds in order to contribute to the valorization of medicinal plants in the Central African Republic. Polyphenols, tannins, flavonoids, anthocyanins, and sugars were measured in these plants. They are quantified for the first time from extracts of *M. mabokeensis*. Analysis of the essential oil of *M. mabokeensis* bark by GC-MS and GC-FID identified and quantified 23 volatile compounds. Qualitative analysis of the extracts by GC-MS led to 14 compounds identified in the extracts of this plant. Two molecules of those identified in the extracts contain aromatic nuclei and are known for their pharmacological properties: catechin and phenol,2-(1,1-dimethylethyl)-6-methyl-. *In vitro*, antioxidant, inhibition of 15-LOX, AChE, XOD, and cytotoxic (against human cancer lines of IGROV and OVCAR) biological activities of the extracts of this plant were studied for the first time. Thus, the correlations between the biological activities and the chemical composition of the extracts of the plant studied were obtained. Therefore, original results were obtained for the phytochemical study, the chemical composition of the essential oil, and the biological activities of *M. mabokeensis*.

## Figures and Tables

**Table 1:** Chemical compositions of *M. mabokeensis* Aubrév bark extracts.

Extract	Yields (%)	TPC	TFC	CTC	TAC	Sugars
		mg GAE/g dr	mg QE/g dr	mg CE/g dr	mg C3GE/g dr	mg GAE/g dr
CYHA	1.7	0.9 ± 0.1^c^	nd	nd	0.03 ± 0.0^a^	nd
DCM	0.8	10.2 ± 0.9^c^	nd	2.0 ± 0.6^c^	nd	nd
EtOAc	1.2	354.2 ± 2.7^a^	8.0 ± 0.3^a^	9.9 ± 0.6^a^	nd	78.7 ± 0.2^b^
MeOH	25.0	230.0 ± 3.2^b^	7.0 ± 0.2^b^	7.3 ± 0.3^b^	nd	101.1 ± 0.2^a^

nd: not detected; dr: dry residue. Different letters in the same column indicate significant differences according to Tukey's test (*p* ≤ 0.05).

**Table 2:** Chemical composition of *M. mabokeensis* Aubrév bark essential oil.

No.	RI	Compounds	% area	Class
**1**	950	*α*-Fenchene	0.81	Monoterpene hydrocarbon
**2**	1000	2-Carene	0.41	Monoterpene hydrocarbon
**3**	1026	*p*-Cymene	0.53	Monoterpene hydrocarbon
**4**	1029	Sylvestrene	0.80	Monoterpene hydrocarbon
**5**	1032	Eucalyptol	1.56	Monoterpene oxygenated
**6**	1091	2-Nonanone	1.09	Other
**7**	1150	Camphene hydrate	0.47	Monoterpene oxygenated
**8**	1178	(E,E,Z)−1,3,5,8-Undecatetraene	1.62	Other
**9**	1337	*δ*-Elemene	1.70	Sesquiterpene hydrocarbon
**10**	1366	Cyclosativene	6.54	Sesquiterpene hydrocarbon
**11**	1408	*α*-Gurjunene	7.39	Sesquiterpene hydrocarbon
**12**	1429	*cis*-Thujopsene	4.79	Sesquiterpene hydrocarbon
**13**	1440	*β*-Humulene	0.93	Sesquiterpene hydrocarbon
**14**	1473	*γ*-Gurjunene	3.02	Sesquiterpene oxygenated
**15**	1503	2,6-Dibutyl-4-me-phenol	1.13	Sesquiterpene oxygenated
**16**	1535	8,14-Cedranoxide	18.88	Sesquiterpene oxygenated
**17**	1569	Caryophylleneoxide	5.86	Sesquiterpene oxygenated
**18**	1602	10-Epi-*γ*-eudesmol	5.84	Sesquiterpene oxygenated
**19**	1624	Dillapiole	3.85	Sesquiterpene oxygenated
**20**	1655	14-Hydroxy-1-epi-caryophyllene	2.76	Sesquiterpene oxygenated
**21**	1682	*cis*-*α*-Santalol	1.60	Sesquiterpene oxygenated
**22**	1929	Methyl palmitate	1.23	Other
**23**	2113	Phytol	27.19	Other
**Total identified**			99.92	
Monoterpene hydrocarbons			2.55	
Monoterpene oxygenated			2.03	
Sesquiterpene hydrocarbons			21.27	
Sesquiterpene oxygenated			42.94	
Others			31.13	

**Table 3:** GC-MS compounds detection and identification in different extracts of *M. mabokeensis* Aubrév before and after the derivatization.

No.	Rt (min)	Compound	CYHA	DCM	EtOAc	MeOH
Before derivatization						
1	11.33	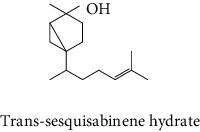	+	nd	nd	nd
2	13.39	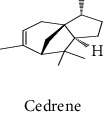	+	nd	nd	nd
3	15.84	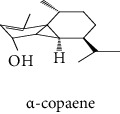	+	+	nd	nd
4	15.99	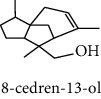	nd	+	nd	nd
**After derivatization**						
1		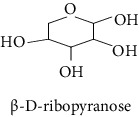	nd	nd	nd	+
2	13.39	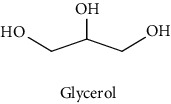	nd	nd	+	nd
3	15.84	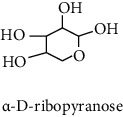	nd	nd	+	nd
4	15.99	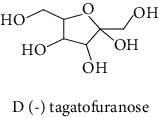	nd	nd	nd	+
5	17.11	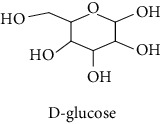	nd	nd	nd	+
6	17.56	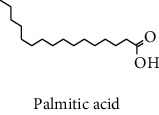	+	nd	nd	nd
7	18.63	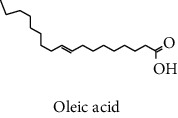	+	nd	nd	nd
8	20.24	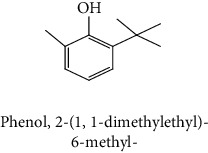	+	+	nd	nd
9	21.82	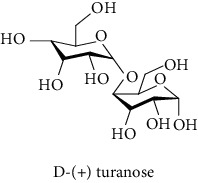	nd	nd	nd	+
10	24.84	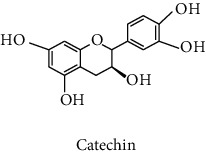	nd	nd	+	+

**Rt:** retention time; **nd:** not detected; **CYHA**: cyclohexane; **DCM**: dichloromethane; **EtOAc**: ethyl acetate; **MeOH**: methanol.

**Table 4:** Phenolic compounds identified in the different extracts of *Manilkara mabokeensis* Aubrév bark by HPLC- DAD.

No.	Rt (min)	Compounds	Concentration (mg/g dr)				References
			CYHA	DCM	EtOAc	MeOH	
**1**	2.20	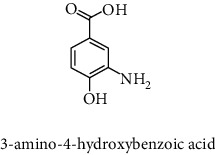	nd	nd	nd	0.1	[19]
**2**	19.19	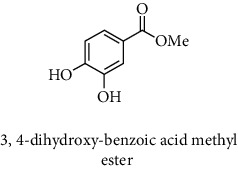	nd	nd	61.8	1.9	[20]
**3**	46.13	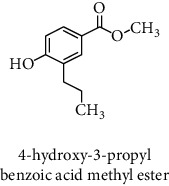	0.3	0.3	nd	0.3	[21]
**4**	47.94	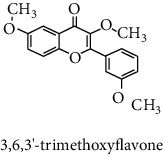	nd	0.1	nd	nd	[22]

**Rt:** retention time; **nd:** not detected; **CYHA**: cyclohexane; **DCM**: dichloromethane; **EtOAc**: ethyl acetate; **MeOH**: methanol.

**Table 5:** Correlation matrix (Pearson (n)).

Variables	TPC	TFC	CTC	AChE	15-LOX	XOD	IGROV	OVCAR	DPPH	TAC
TPC	1	0.98	0.99	0.61	0.59	0.56	0.94	−0.43	−0.51	−0.57
TFC	0.98	1	0.97	0.69	0.74	0.71	0.91	−0.45	−0.55	−0.57
CTC	0.98	0.97	1	0.72	0.59	0.59	0.86	−0.57	−0.38	−0.70
AChE	0.61	0.69	0.72	1	0.66	0.77	0.32	−00.89	0.11	−0.91
15-LOX	0.59	0.74	0.59	0.66	1	0.98	0.55	−0.26	−0.59	−0.31
XOD	0.56	0.71	0.59	0.77	0.98	1	0.46	−0.42	−0.42	−0.45
IGROV	0.94	0.91	0.86	0.32	0.55	0.46	1	−0.09	−0.75	−0.24
OVCAR	−0.43	−0.45	−0.57	−0.90	−0.26	−0.42	−0.10	1	−0.48	0.99
DPPH	−0.51	−0.55	−0.38	0.11	−0.59	−0.42	−0.75	−0.48	1	−0.36
TAC	−0.57	−0.58	−0.70	−0.91	−0.31	−0.45	−0.24	0.99	−0.36	1

**Table 6:** Anti-15-LOX, anti-AChE, and anti-XOD activities of *M. mabokeensis* Aubrév bark extracts (50 *μ*g/mL).

Extracts	Anti-15-LOX (%)	Anti-AChE (%)	Anti-XOD
CYHA	13.0 ± 0.3^c^	na	na
DCM	na	43.5 ± 0.1^b^	na
EtOAc	25.7 ± 0.1^b^	46.5 ± 0.1^b^	11.1 ± 1.3^b^
MeOH	65.8 ± 0.1^a^	71.0 ± 0.2^a^	41.5 ± 4.8^a^
NDGA	95.3 ± 0.2	—	—
Galanthamine	—	95.9 ± 0.2	—
Allopurinol	—	—	75.5 ± 0.9

na: not active. Different letters indicate significant differences according to Tukey's test (*p* ≤ 0.05).

**Table 7:** Cytotoxic activity of *M. mabokeensis* Aubrév bark extracts (50 *μ*g/mL) against IGROV and OVCAR cell lines.

Extracts	IGROV (%)	OVCAR (%)
CYHA	16.3 ± 3.0^c^	49.5 ± 6.5^a^
DCM	na	15.3 ± 3.0^d^
EtOAc	48.7 ± 2.6^a^	21.7 ± 4.9^b^
MeOH	30.7 ± 5.7^b^	17.9 ± 4.3^c^
Tamoxifen	77.4 ± 7.6	

na: not active. Different letters indicate significant differences according to Tukey's test (*p* ≤ 0.05).

**Table 8:** Correlations between variables and factors.

	F1	F2
TPC	0.92	−0.19
TFC	0.97	−0.19
CTC	0.95	−0.03
AChE	0.84	0.50
15-LOX	0.79	−0.25
XOD	0.81	−0.06
IGROV	0.78	−0.52
OVCAR	−0.61	−0.79
DPPH	−0.40	0.91
TAC	−0.71	−0.69

## Data Availability

The data used to support findings of this study are included within the article.

## Abbreviations

15-LOX: 15-Lipoxygenase
ACTHi: Acetylthiocholine iodide
AMDIS: Automated mass spectral deconvolution and identification system
CTC: Condensed tannin content
dr: Dry residue
CYHA: Cyclohexane
DCM: Dichloromethane
DMEM: Dulbecco's modified Eagle's medium
DMSO: Dimethyl sulfoxide
DNS: 3,5-Dinitrosalicylic acid
DTNB: Dithiobisnitrobenzoate
EtOAc: Ethyl acetate
FID: Flame ionization detector
GC-MS: Gas chromatography-mass spectrometry
HPLC: High-performance liquid chromatography
MeOH: Methanol
MTT: 3-(4,5-Dimethylthiazol-2-yl)-2,5-diphenyltetrazolium bromide
Na_3_PO_4_: Sodium phosphate
NDGA: Nordihydroguaiaretic acid
NIST: National Institute of Standards and Technology
OVCAR: Human ovarian cancer
TAC: Total anthocyanins content
TFC: Total flavonoids content
TPC: Total phenolic content.
